# The Typical Damage Form and Mechanism of a Railway Prestressed Concrete Sleeper

**DOI:** 10.3390/ma15228074

**Published:** 2022-11-15

**Authors:** Ruilin You, Jijun Wang, Na Ning, Meng Wang, Jiashuo Zhang

**Affiliations:** 1 Railway Engineering Research Institution, China Academy of Railway Sciences Corporation Limited, Beijing 100081, China; 2 School of Civil Engineering, Universiti Sains Malaysia, Pinang 14300, Malaysia

**Keywords:** concrete sleeper, damage form, damage mechanisms, longitudinal crack, transversal crack, limit state method

## Abstract

Prestressed concrete sleepers are an important track component that is widely used in railway ballast track. Prestressed concrete sleepers have high strength, strong stability, and good durability; thus, their operation and use in railways are beneficial. However, in different countries and regions, certain damage to sleepers typically appears. Existing research on concrete sleepers focuses primarily on the structural design method, the application of new materials, theoretical analysis, and bearing strength test research, while ignoring sleeper damage. There are a few sleeper damage studies, but they look at only one type of damage; thus, there is no comprehensive study of prestressed concrete sleeper damage. The damage forms of prestressed concrete sleeper damage are thus summarized in this study, and the theory of the causes of prestressed concrete sleepers is analyzed based on the limit state method for the first time. The findings indicate that sleeper structure design is the primary cause of its operation and use status, and that special measures should be considered based on sleeper use conditions. In addition to meeting design requirements, materials, curing systems, product inspection, and other factors must be considered during manufacturing to improve the sleepers’ long-term performance. Keeping the track in good condition, including but not limited to the state of fasteners, ballast bed, and track geometry is also an important aspect of preventing sleeper damage. The outcomes of this study provide better insights into the influences of damage to railway prestressed concrete sleepers and can be used to improve track maintenance and inspection criteria.

## 1. Introduction

Railways are one of the most important modes of transportation for both passengers and freight worldwide. Railway traffic is increasing due to the widespread use of high-speed lines, and new railway lines are being built in many countries. Parallel to these developments, the production and the use of railway sleepers are increasing. Railway sleepers are key components in railway tracks that carry the loads transferred from vehicles to the rails, support the rails, protect the gauge, and withstand horizontal and vertical rail movement [1,2,3,4]. Sleepers can be made from a variety of materials, including wood, concrete, steel, and composites [5,6,7,8,9].

The use of prestressed concrete sleepers has grown in recent years due to their high quality. Furthermore, prestressed concrete sleepers are more environmentally friendly than creosote-treated wooden sleepers [5,8,10]. Currently, approximately 500 million prestressed concrete sleepers are required every year in railway networks all over the world [11].

Most prestressed concrete sleepers are in good operating condition, but some are damaged in different ways [12,13]. Many studies have been performed on prestressed concrete sleepers, primarily focusing on dynamic load [14,15], bearing capacity [10,16], and structural design [17,18]. However, systematic research on the damage form and mechanism of railway prestressed concrete sleepers is currently limited.

Berntsson and Chandra investigated calcium chloride damage in concrete sleepers and concluded that calcium chloride is harmful to concrete [19]. Pawluk et al. researched the durability of prestressed concrete sleepers [20]. Ravindrarajah and White investigated the effect of non-delayed heat application on prestressed concrete sleeper strength [21]. Shojaei et al. conducted a study on the application of alkali-activated slag (AAS) concrete in the production of prestressed reinforced concrete sleepers [22]. Rezaie et al. performed a study on the factors affecting longitudinal crack propagation in prestressed concrete sleepers [23]. Zeman et al. investigated the mechanism of rail-seat abrasion of prestressed concrete sleepers in North America [24]. Zakeri et al. study on the variation of loading pattern of concrete sleeper due to ballast sandy contamination in sandy desert areas and the failures of railway concrete sleepers during service life [25,26]. These studies only analyze a single type of damage in prestressed concrete sleepers, but the damage mechanism and analysis methods were not investigated. to date. Thus, the various types of damage in prestressed concrete sleeper damage are summarized in detail in this study. In addition, for the first time, the theory of the causes of prestressed concrete sleepers is analyzed using the limit state method. The findings of this study provide a clearer picture of the effects of damage to railway prestressed concrete sleepers and improve track maintenance and inspection criteria.

## 2. Typical Form and Influence of Prestressed Concrete Sleeper Damage

### 2.1. Transverse Cracks

Transverse cracks are perpendicular to the long axis of the concrete sleeper. Because of the change in the ballasted bed support state and the randomness of the trainload, the concrete sleeper may experience a load-bending moment that exceeds its strength during service, resulting in transverse cracks. According to the appearance position, the transverse crack of the concrete sleeper can be classified into two types: a transverse crack of the rail seat section and a transverse crack of the central section.

**Figure 1:**
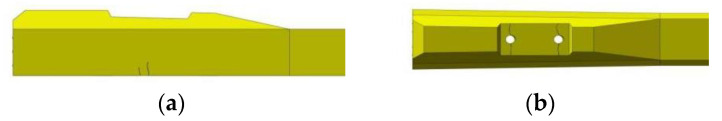
Transverse crack in a rail seat section: (**a**) lower part crack in the rail seat section; (**b**) upper part crack in the rail seat section.

The transverse crack of the rail seat section can be divided into the lower part crack and upper part crack, as shown in Figure 1; these cracks are caused by the positive and negative bending moments of the lower section of the sleeper exceeding its bearing strength, respectively. Typically, the width of the transverse crack is narrow, but if the load bending moment is too large, a crack will develop, and the sleeper will fail.

**Figure 2:**
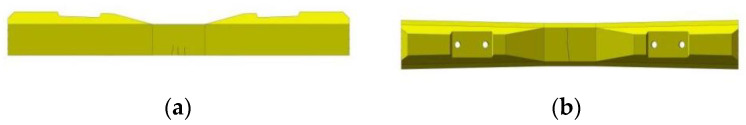
The transverse crack of the central section: (**a**) lower part crack of the central section; (**b**) upper part crack of the central section.

A transverse crack of the central section can be divided into a lower part crack and upper part crack, as shown in Figure 2. These cracks are caused by the positive and negative bending moments of the lower section of the sleeper exceeding its bearing strength, respectively. Typically, cracks in the upper part of the central section of the concrete sleeper due to the negative moment being too large are more common.

### 2.2. Longitudinal Crack

**Figure 3:**
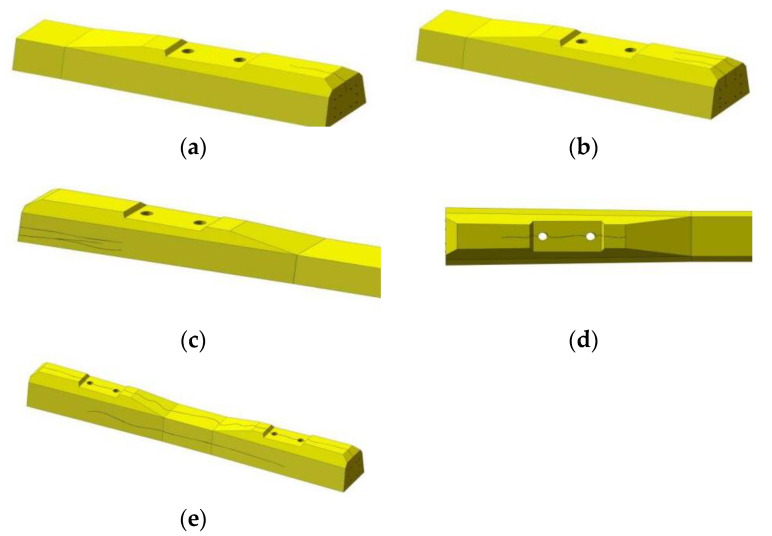
Longitudinal crack: (**a**) end surface crack; (**b**) longitudinal cracks on the upper surface of the end part (**c**) longitudinal crack at the embedded parts; (**d**) horizontal and longitudinal crack on the side; (**e**) the through longitudinal crack of the sleeper.

Cracks along the long axis of the sleeper are collectively referred to as longitudinal cracks. For the prestressed concrete sleeper, stress was applied to the concrete in the design, manufacturing, construction and maintenance links of improper treatment, which may lead to the occurrence and development of longitudinal cracks. A longitudinal crack in a sleeper is generally categorized as an end surface crack, a longitudinal crack on the upper surface, a horizontal longitudinal crack on the side, a longitudinal crack at the embedded parts and a longitudinal crack through the sleeper (as shown in Figure 3).

Longitudinal cracks in prestressed concrete sleepers have long been a source of concern. After the longitudinal crack appears, the sleeper’s strength and durability decrease, as does its ability to maintain track geometry; thus, longitudinal cracks must be prioritize during maintenance work.

### 2.3. Inclined Crack

**Figure 4:**
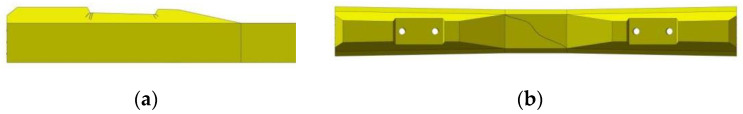
Inclined crack: (**a**) inclined crack at the base corner of the sleeper shoulder; (**b**) inclined crack in the middle of the sleeper.

Inclined cracks on the sleeper’s surface become oblique cracks and typically appear at the bottom corner of the retaining shoulder or on the surface of the sleeper, as shown in Figure 4. The former is generally caused by the excessive shear load of the sleeper, while the latter may be caused by improper tamping and repair operations in the maintenance process.

### 2.4. Map-Like Crack

**Figure 5:**
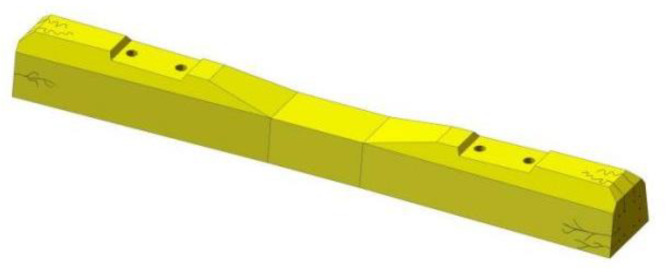
Map-like crack of a concrete sleeper.

An irregular mesh of cracks on the surface of a sleeper is also called sleeper map-like cracks, as shown in Figure 5. Typically, a sleeper map-like crack is related to the materials, the manufacturing process and the environmental conditions used of the concrete sleeper. When a map-like crack appears in the early stage of sleeper curing process, the crack width is small and generally appears on the sleeper’s surface. With the extension of the width and range of the crack, it will continue to develop and eventually lead to the sleeper’s life being markedly reduced.

### 2.5. Rail Seat Abrasion

**Figure 6:**
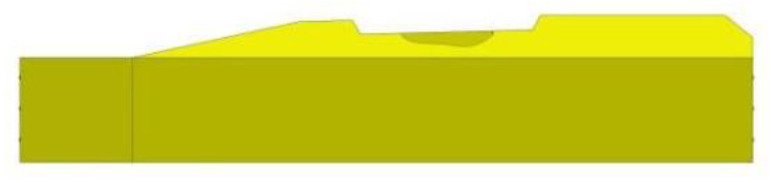
Rail seat abrasion of the concrete sleeper.

Abrasion refers to the use of a sleeper during interaction with the connected rail parts for a long time, resulting in damage to the connection site. The abrasion form of the sleeper is primarily the wear of the rail seat area, as shown in Figure 6, which is primarily caused by the long-term impact friction between the under-rail pad and the bearing surface. The geometry of the track is altered and the bearing capacity of the sleeper is weakened by the abrasion of the rail seat region, which worsens the state of the railway track.

### 2.6. Break Damage and Block Dropped

**Figure 7:**
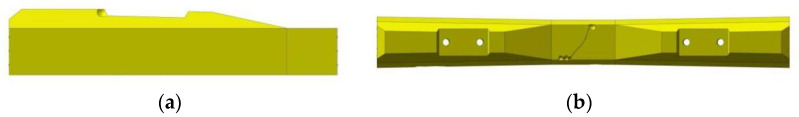
Break damage and block dropped: (**a**) block dropped off the shoulder; (**b**) break damage in the middle part of the sleeper.

Break damage and drop blocks generally appear in the shoulder and the upper surface of the concrete sleeper, as shown in Figure 7. Typically, breaks and drops are caused by external loads that are too large, such as in the process of transportation and unloading and the process of trackbed tamping. The accidental improper external loads will also lead to the sleeper knock block in the small radius curve section because the large lateral load of the train will also lead to the phenomenon of shoulder damage. The stress condition of the sleeper, which is vulnerable to stress concentration and other damage, will get worse with loss and block drop, and the stability of the track construction will also suffer.

### 2.7. Comprehensive Injury

Sleeper damage to a railroad track often consists of two or more types of damage occurring at the same time rather than just one. For instance, the simultaneous appearance of a transverse fracture and collapse, a longitudinal crack and a surface crack, and a shoulder oblique crack and knock are frequent. Different types of harm will result from the sources of these simultaneous incidences of injury, or the “inducing variables.” On the other hand, one kind of damage might potentially cause another kind of harm to occur. The bearing capacity of the sleeper and the stability of the track structure are more severely affected when various types of damage take place at the same time. It is important to identify the precise causes of damage during maintenance and to suggest early preventive solutions.

## 3. Analysis of the Damage Mechanism of Prestressed Concrete Sleepers

### 3.1. Analysis Method

The essence of the damage of a prestressed concrete sleeper is that the external load effect exceeds its resistance; thus, damage analysis can be evaluated using the limit state method.

The limit state method is commonly used in the structure design. This method uses failure probability or a reliable index to measure structure reliability and to establish a relationship between the structure limit equation of state and the probability theory of structural reliability [27]. When designed by the limit state method, the structural resistance uses the strength R of the material multiplied by a load factor; this value shall not be any less than the total load effect of each load effect S of the structure multiplied by the respective load factor S, as shown in Equation (1):

Σ(γ · S) ≤ ΦR(1)

**Figure 8:**
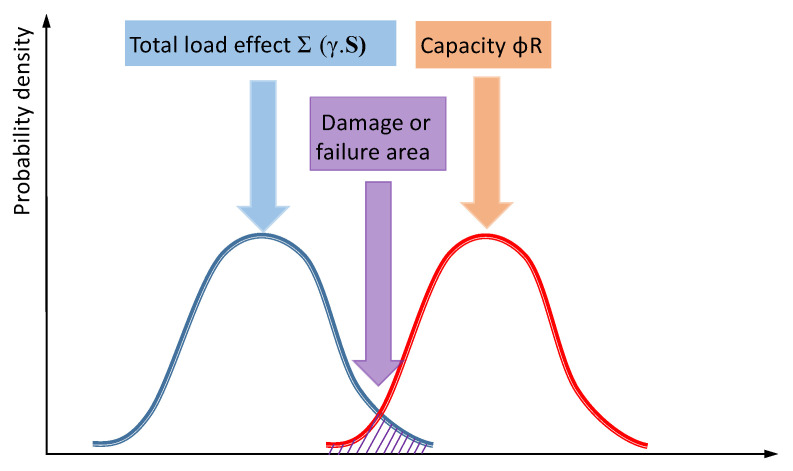
Schematic diagram of the distribution curve of sleeper damage or failure probability.

The damage phenomenon of prestressed concrete sleepers is contrary to the working condition considered in the design process due to the external load effect exceeding its resistance; however, the concept of its analysis is the same. The probability distribution curve of the total load effect, structural resistance and sleeper damage failure is shown in Figure 8. Therefore, the damage mechanism of prestressed concrete sleepers can be analyzed from the total load effect, internal resistance and comprehensive factors.

### 3.2. External Load Effect

#### 3.2.1. Design Load Effect

When designing a prestressed concrete sleeper, the load moment produced by the train dynamic load in the sleeper is primarily considered. The designed load moment primarily includes the lower section of the sleeper and the positive load bending moment of the section in the sleeper.

Because the load bending moment of the sleeper is directly related to the dynamic load of the train and the support state of the sleeper itself, if the above factors exceed the design range, an excessive load bending moment will lead to sleeper damage.

**Figure 9:**
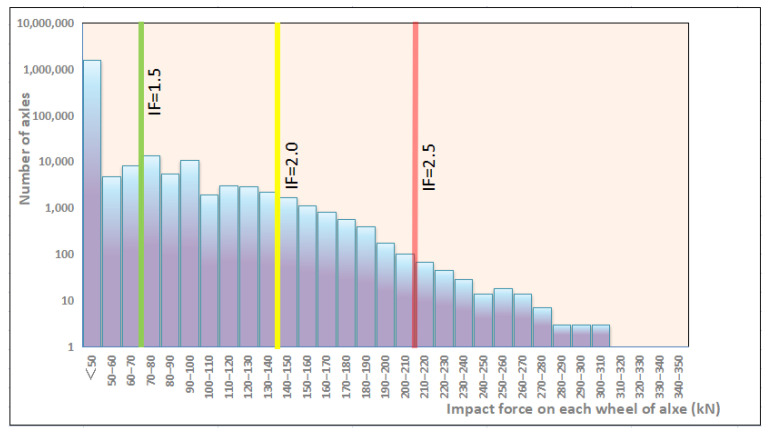
Typical impact force statistical data on the track at Braeside. Note: Impact factor (IF) = 1+ (impact force)/(static wheel load).

Queensland University of Technology in Australia tested the impact force of two separate sites, Braeside and Raglan [10,17]. At these two sites, the maximum static axle load of the operational vehicles is 28 tons. The field measurement data in Braeside is shown in Figure 9. The trainload of the sleeper has a wide fluctuation range.

**Figure 10:**
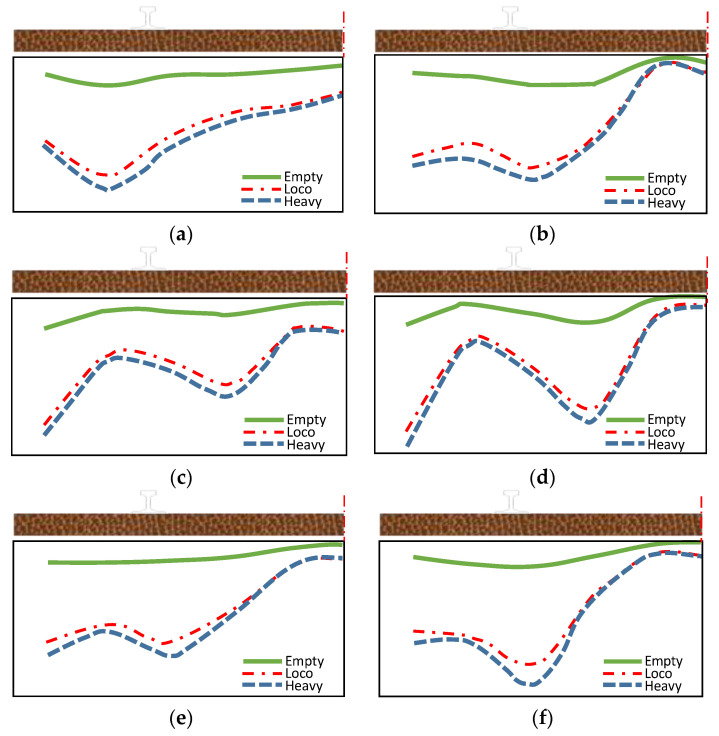
Support conditions experimentally measured in the field. (**a**) moderate ballast 1; (**b**) moderate ballast 2; (**c**) new ballast 1; (**d**) new ballast 2; (**e**) fouled ballast 1; (**f**) fouled ballast 2.

The bottom compression stress of the sleeper in various bed states is varied according to the measured findings of the compression stress at the bottom of the sleeper in those beds [26,28]; thus, the load-bending moment of the sleeper will differ appropriately., as shown in Figure 10.

#### 3.2.2. Environmental Effect

Environmental utility primarily refers to the external environmental effects of moisture, temperature, chemical erosion, and freezing and thawing during the operation of the concrete sleeper. The AAR and DEF that often occur in concrete sleepers are typical damages caused by environmental effects.

##### Alkali-Aggregate Reaction

**Figure 11:**
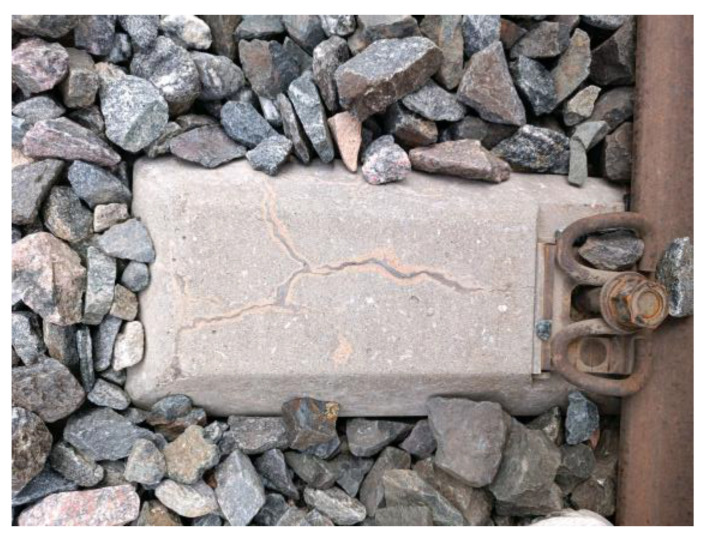
AAR damaged the concrete sleeper. Note: These images also show other factors in addition to AAR.

The alkaline-aggregate reaction (AAR) refers to the concrete hole solution by cement or alkali admixture, mineral admixture, and the environment released Na^+^, K^+^, OH^−^ and the aggregate of harmful active minerals in the expansion reaction, resulting in concrete expansion and cracking phenomenon [29]. Figure 11 shows the concrete sleeper’s AAR damage.

There are three conditions for AAR occurrence:

- The aggregate in the concrete has alkali activity;
- There is a certain amount of soluble alkali in concrete;
- Sufficient water or wet environmental conditions are present.

The alkali-aggregate reaction can primarily be divided into two types of reaction types: alkali-silicic acid reaction (ASR) and alkali-carbonate reaction (ACR).

ASR is the chemical reaction between the alkali in concrete and the active SiO_2_ in the aggregate to form the alkali silicate gel. The gel volume is greater than the SiO_2_ volume before the reaction (as shown in Equation (2)). The gel water absorption causes concrete expansion and cracking, and the internal reaction mechanism is shown as follows. The ASR generally has a large swelling area that accounts for cracking within 10–20 years after the construction of the concrete structure and can further develop damage to the entire structure:

Na^+^(K^+^) + SiO_2_ + OH^−^ → Na(K) – Si − Hgel (2)

ACR is the reaction of the alkali in the concrete with the dolomite crystals contained in the active carbonate aggregate, producing concrete expansion and cracking, as shown in Equations (2) and (3). Studies have shown that LiOH can be used to distinguish ASR from ACR. The ACR reaction development speed is fast, and the general concrete project is built in 2~3 years of expansion and cracking, and cannot be repaired and reinforced:

CaMg(CO_3_)_2_ + 2ROH = Mg(OH)_2_ + CaCO_3_ + R_2_CO_3_ (3)

R_2_CO_3_ + Ca(OH)_2_ = 2ROH + CaCO_3_ (4)

##### Delayed Ettringite Formation

**Figure 12:**
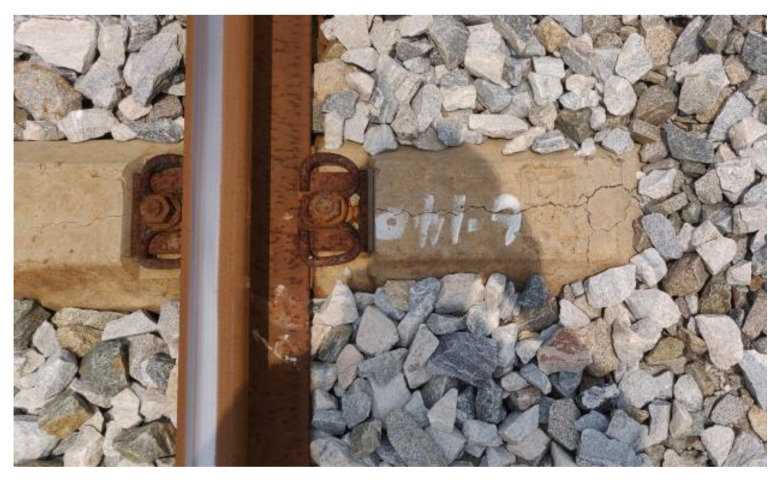
DEF damaged the concrete sleeper. Note: These images also show other factors in addition to DEF.

The Delayed Ettringite Formation (DEF) is a form of sulfate erosion in cement concrete with sulfate ions from the inside of cement concrete. Therefore, the definition of delayed alum rock can be considered as follows: in hardened cement concrete, not from the process of sulfate outside the cement concrete, the harm caused by delayed alum rock often appears only months or years later. The delay of slurry expansion caused by ettringite formation will cause cracks in the interface between the cement slurry and aggregate-cement slurry [30], which leads to cracks in the concrete sleeper (as shown in Figure 12).

Results show that the primary characteristics of delayed calcium generation are as follows:

- Typically, delaying the formation of ettringite occurs when the cement concrete experiences a temperature of 70 °C and causes serious expansion cracking in an environment of high relative humidity. Between 70 °C and 100 °C, the higher the temperature is, the more severely inflated;
- The expansion and development of cement concrete in wet air are slower than those in water, but its macrocracks are larger;
- The expansion caused by delayed ettringite begins from the outside of the cement concrete and gradually expands to the inside;
- The expansion caused by delayed calcium ettringite is affected by the nature and size of the aggregate used;
- When the concrete structure has been damaged, delayed calcium damage is typically accompanied by an AAR.

There are still some disputes about the mechanism of DEF caused by concrete structure damage, which primarily includes the following aspects. First, a real concrete structure engineering environment is diverse, and the causes of concrete damage are complex. For example, AAR consumes the alkali in the liquid phase, which causes the solubility of calcium in the liquid phase to decline and precipitate out. Therefore, when concrete experiences cracking failure, it is often considered the joint action of AAR and DEF, and the reaction of AAR occurs before DEF. In addition, other factors can also affect DEF, which cannot show whether the primary cause of concrete destruction is DEF. Second, calcium ettringite is widely used as an indispensable component of concrete shrinkage compensation and cement early strong and fast hard, even if the discovery of calcium ettringite cannot prove its real impact on the performance of concrete.. Third, laboratory small size test blocks can be observed in the water after several days of the new formation of ettringite, marginally larger mortar test blocks can see the delayed formation of ettringite, and the real engineering of large size concrete to several years to see this phenomenon. Therefore, the action mechanism and destruction process must be investigated in more detail.

#### 3.2.3. Accidental Load Effect

**Figure 13:**
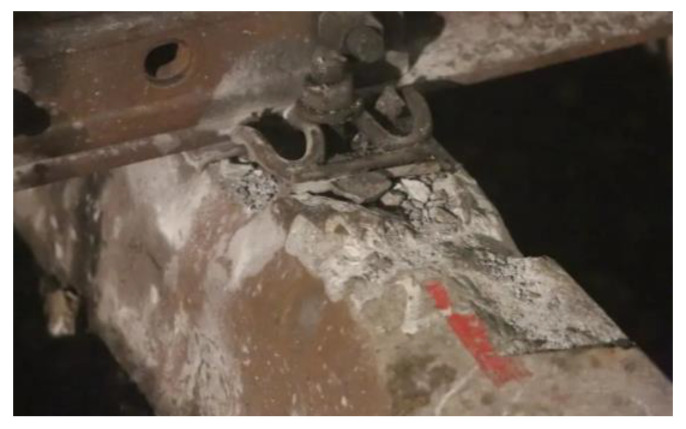
Damaged concrete sleeper in the derailment.

**Figure 14:**
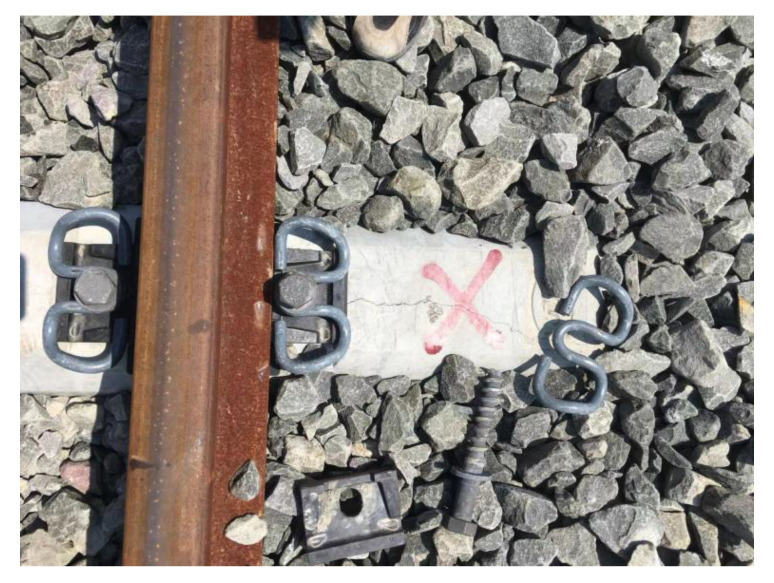
Excessive torque of the buckling bolt causes sleeper damage. Note: These images also show other factors in addition to the torque of the buckling bolt.

**Figure 15:**
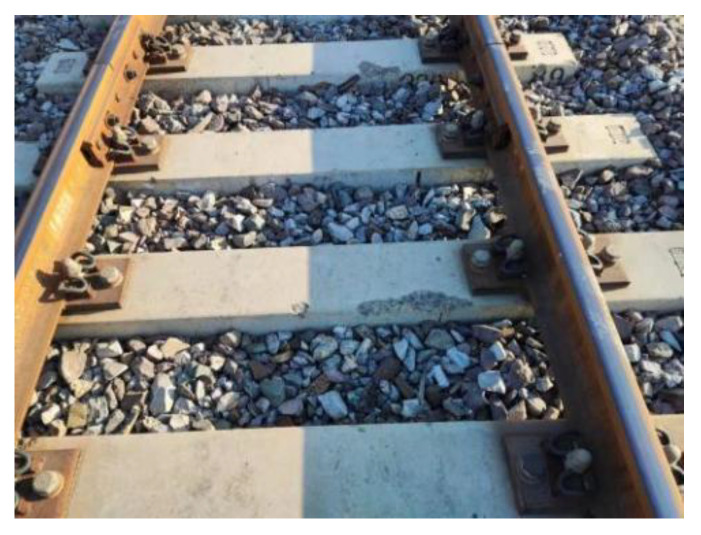
Impact load caused damage during construction of the sleeper.

The load effect under accidental conditions must be taken into account in the sleeper design in addition to bearing the operating load of the design. In rare circumstances, these unintended load effects can harm the sleeper. When a train is derailed [31], the sleeper receives a very large impact load and may become damaged or even fail, as shown in Figure 13. Excessive bolt torque on the fasteners can also cause damage during sleeper laying and maintenance [32] (as shown in Figure 14). During sleeper laying and maintenance, the sleeper can also be knocked and damaged, as shown in Figure 15.

For sleeper damage with an accidental load effect, the proportion of damaged sleepers is not large, and the characteristics are important. Combined with the situation on the site, it is easier to judge the cause of the damage.

#### 3.2.4. Poor Track Structure Status

**Figure 16:**
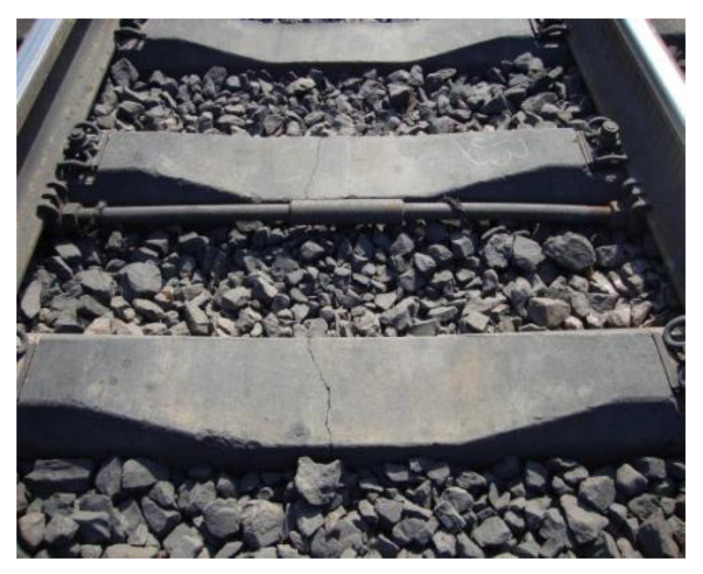
Track bed hardening damages the concrete sleeper.

**Figure 17:**
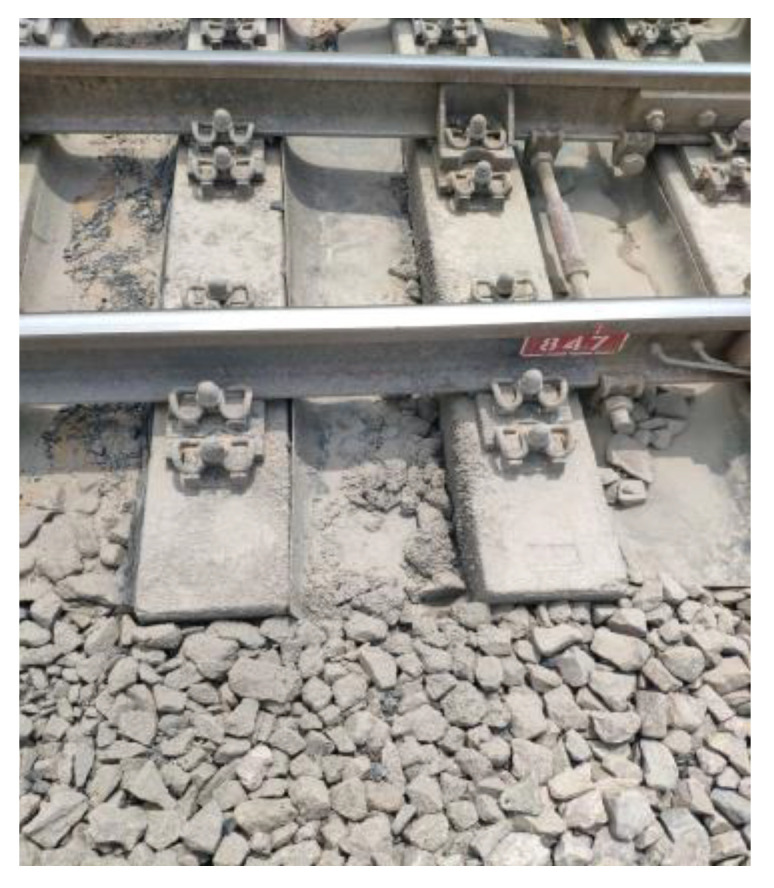
Track bed frost boiling damages the concrete sleeper.

Sleepers are a track component in the ballast track structure when the track structure is in poor condition (e.g., the line appears empty hanging phenomenon, trackbed hardening, trackbed frost boiling) will also lead to sleeper damage or even failure, as shown in Figure 16 and Figure 17.

**Figure 18:**
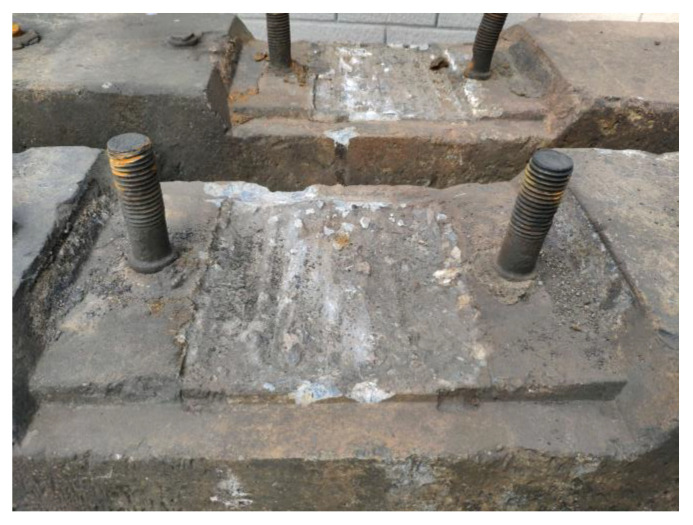
Concrete sleeper rail seat abrasion.

Sleeper damage and poor railway conditions have an interaction. Inadequate railroad conditions can cause sleeper damage, and in the other direction, poor track conditions can be made worse by sleeper damage [33]. Examples of this include the mutual impact and aggravation between the track’s geometric condition and the wear surface of the sleeper, as shown in Figure 18. Therefore, sections with poor track conditions should be rapidly repaired during track structure operation to prevent further degradation of the track state and escalation of track component damage. 

### 3.3. Structural Self-Resistance

#### 3.3.1. Structural Design Strength

When prestressed concrete sleepers are used in a track design, if the strength is insufficient, damage will occur during its service life. For one type of sleeper, the same damage form usually occurs in different lines, different manufacturers and under similar operating conditions. The design strength of the sleeper structure is insufficient; typically, the reasons for this phenomenon are a small concrete sleeper section, an unreasonable prestressed steel wire configuration, insufficient configuration or no stirrups.

**Figure 19:**
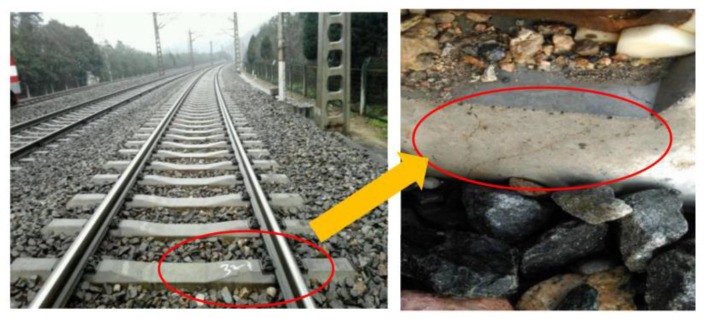
Transverse crack in the rail seat section of the concrete sleeper.

**Figure 20:**
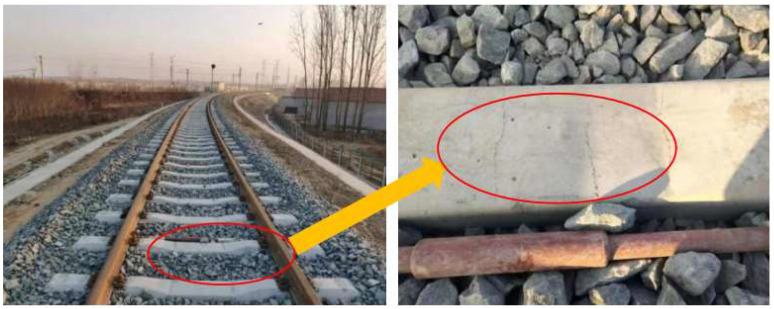
Transverse crack in the central section of the concrete sleeper.

**Figure 21:**
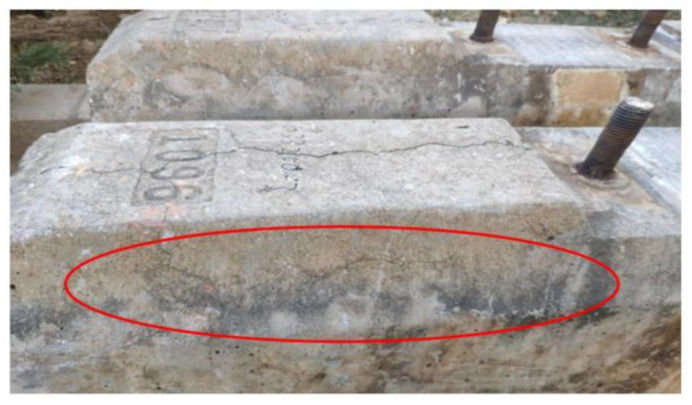
Horizontal crack on the side of the concrete sleeper.

Typical sleeper damage caused by insufficient structural design strength is a transverse crack at the rail seat section, as shown in Figure 19, where a transverse crack in the sleeper (Figure 20) and a horizontal crack on the side of the sleeper occur (Figure 21).

#### 3.3.2. Manufacturing Quality

In addition to the structural design leading to the insufficient structural resistance of prestressed concrete sleepers, the production quality also has an important impact on the structural resistance of sleepers, and the general production quality of sleeper damage will appear in different external load superposition forms. In the production and manufacturing process, the resistance of sleepers is primarily affected by the material quality and curing process.

(1) Raw material quality

Under the action of the sleeper mentioned above, the AAR will lead to damage, which is closely related to the raw material quality of the sleeper. An important indicator of high-quality materials is not to produce or cause AAR damage to concrete. Steps to prevent AAR are typically required to eliminate the reaction:

- Select the inactive aggregate;
- Control the alkali content of cement (Na_2_O+0.658K_2_O), which should not exceed 0.6%;
- Appropriate incorporation of mineral fine admixture reduces the amount of cement to reduce the temperature difference stress in the concrete caused by the heat of cement hydration.

(2) Curing process

**Figure 22:**
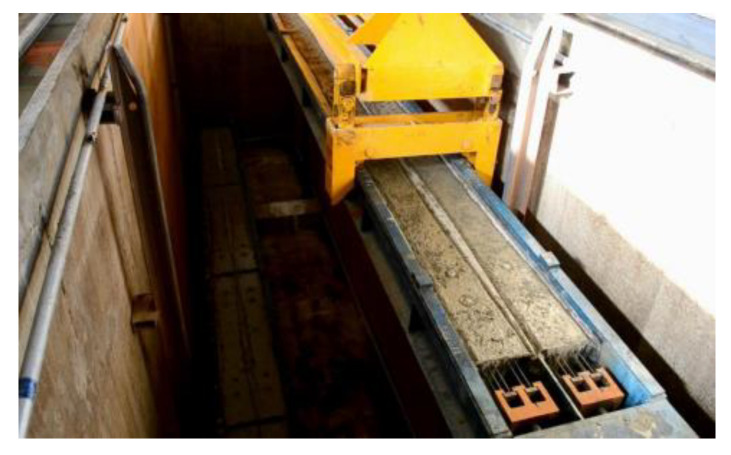
Steam curing of the concrete sleeper.

A prestressed concrete sleeper is typically made using steam curing, as shown in Figure 22. Steam curing is a type of hardening process that accelerates the development of concrete strength. During hardening, the hydration reaction of cement produces a large amount of hydration heat. Sleeper curing in the early stage is the most important period of health preservation. If the temperature in the curing pool is high, the temperature in the pool is higher than that inside the sleeper, and thermal hydration accumulation inside the pool is not easy to distribute. The gas and moisture inside the concrete mixture are heated and expanded. At this time, the concrete strength is low, and there is no resistance. However, internal stress causes concrete deformation and can even produce microfine cracks. This situation will lead to lower sleeper structural resistance. Thus, damage during operation affects a sleeper’s service life. A reasonable sleeper curing system includes the following points:

- Determine the relationship between the temperature of the sleeper concrete and the surrounding air temperature throughout the process of the sleeper life cycle, and avoid a large temperature difference between the temperature of the sleeper concrete and the surrounding air temperature;
- The temperature change of the sleeper core should not exceed 15 °C/h, and the cooling rate should not exceed 15 °C/h;
- The difference between the temperature of the sleeper surface and the outside environment when leaving the curing pool should not exceed 15 °C.

### 3.4. Multiple Factors

The external load effect and the structure’s resistance are used to analyze the primary causes of prestressed concrete sleeper damage. However, the causes of sleeper damage during operation are frequently caused by multiple factors rather than a single factor. For example, the quality control of sleeper manufacturing lax alkali activity of materials exceeds the standard. This sleeper is used in a humid environment and certain acidic medium, and it is likely to exhibit sleeper surface cracking damage caused by AAR, which is the comprehensive factor of manufacturing quality and environmental effect.

In addition, the comprehensive sleeper damage mentioned above can also be caused by the comprehensive superposition of multiple factors.

## 4. Conclusions

This paper summarizes the damage forms of concrete sleepers and analyzes the damage mechanism of prestressed concrete sleepers from the three aspects of the external load effect, structural self-resistance and comprehensive factors. Based on the results of this paper, prestressed concrete sleepers can prevent damage and improve the quality level from the following aspects:

(1) Structural design guarantee of prestressed concrete sleeper

Sleeper structure design is the fundamental cause of its operation and use status. An excellent structural design can avoid damage to a large extent and should consider the appearance size, reinforcement design, manufacturing, operation, maintenance systems and environmental conditions of the sleeper. Therefore, the prevention of concrete sleeper damage starts with improving the design quality of the sleeper structure.

(2) Manufacturing quality control

In addition to structural design, the production quality is also an important factor to prevent concrete sleeper damage. During manufacturing, in addition to meeting design requirements, materials, curing systems, product inspection and other aspects must be considered to improve the long-term sleeper performance.

(3) Use of environmental condition adaptability

Temperature, humidity and chemical erosion in the use environment are also important factors that can lead to sleeper damage. Therefore, in the sleeper design process, special measures should be considered based on these use conditions. For example, when used in cold areas, sleeper design must consider the ability to resist freezing and thawing; in acid rain or sea regions, sleeper design must consider resistance to chemicals.

(4) Track status improvement

A concrete sleeper is a portion of the railway track, and the deterioration of the overall state of the track will inevitably lead to a sleeper bearing a greater load effect, which can lead to damage. Therefore, an important aspect of preventing sleeper damage is to keep the track in good condition, including but not limited to the state of fasteners, the ballast bed and track geometry.

## Data Availability

Not applicable.
